# The long-term impact of early treatment of multiple sclerosis on the risk of disability pension

**DOI:** 10.1007/s00415-018-8764-4

**Published:** 2018-02-01

**Authors:** Erik Landfeldt, Anna Castelo-Branco, Axel Svedbom, Emil Löfroth, Andrius Kavaliunas, Jan Hillert

**Affiliations:** 1 Mapi Group, Stockholm, Sweden; 2 Institute of Environmental Medicine, Karolinska Institutet, Nobels väg 13, 17177 Stockholm, Sweden; 3 Novartis, Stockholm, Sweden; 4 Department of Clinical Neuroscience, Karolinska Institutet, Stockholm, Sweden

**Keywords:** Multiple sclerosis, Disability pension, Absenteeism, Social insurance, Survival analysis

## Abstract

**Objective:**

The objective of this retrospective, observational study was to estimate the long-term impact of early treatment of multiple sclerosis (MS) on the risk of disability pension.

**Methods:**

Our cohort comprised patients with MS in Sweden, identified in a nationwide disease-specific register (the Swedish Multiple Sclerosis Registry), who started treatment with a disease-modifying drug (DMD) between January 1, 2002, and December 31, 2012. We analyzed the association between time from onset of MS to treatment initiation and full-time disability pension using survival analysis.

**Results:**

Our sample comprised 2477 patients. Unadjusted Kaplan–Meier failure functions showed that patients who started treatment within six months after onset had a lower risk of disability pension across follow-up compared with patients initiating therapy after 12 months. Outcomes from the univariate Cox proportional hazards model showed that time from onset to treatment initiation (in years) was significantly associated with disability pension (HR 1.03, *p* < 0.001). Outcomes from the multivariable Cox proportional hazards model showed that patients who started treatment within 6 months after onset had, on average, a 36% lower risk (HR 0.74, *p* = 0.010) of full-time disability pension during follow-up compared with patients starting treatment after 18 months when controlling for age, sex, marital status, university education, and prevalent comorbidities.

**Conclusions:**

We show that early treatment with DMDs of MS is associated with a significantly reduced risk of disability pension. Our findings highlight the potential long-term benefits of early treatment of MS and should be helpful to inform ongoing discussion on the optimum medical management of the disease.

**Electronic supplementary material:**

The online version of this article (10.1007/s00415-018-8764-4) contains supplementary material, which is available to authorized users.

## Introduction

In recent years, an extensive body of literature has accumulated with respect to benefits of early treatment of multiple sclerosis (MS) with disease modifying drugs (DMDs). Specifically, research has shown that early treatment in relation to disease onset is associated with significantly improved physical and mental outcomes, including lower relapse rates and lower Expanded Disability Status Scale (EDSS) scores, both in the short- and long-term [1–8]. In accordance with these findings, in several jurisdictions, including the UK [9] and Sweden [10], clinical consensus is now that DMDs should be offered as early as possible in the medical management of MS. However, there is still an ongoing discussion of the timing of treatment initiation [11], in part due to inconclusive results from observational effectiveness studies.

With respect to the benefits of early treatment outside of the clinical trials setting, little is known of the long-term impact on socio-economic outcomes, including labour-force absenteeism. In our previous research, we have shown that patients with MS have elevated levels of sick leave and disability pension up to 15 years before disease diagnosis compared with the general population, highlighting the burden of disease on affected patients and society [12]. The objective of this study was to estimate the long-term impact of early treatment with DMDs on disability pension in patients with MS in Sweden.

## Methods

### Study design and population

This was a retrospective, observational study of the impact of early treatment on disability pension in patients with MS in Sweden. Our sample population was identified through a nation-wide disease-specific registry, the Swedish Multiple Sclerosis Registry [13] encompassing more than 70 care-units in regions across Sweden, and initially comprised all patients with MS that started treatment with a DMD [interferon beta (Avonex, Rebif, Betaferon, and Extavia), glatiramer acetate (Copaxone), natalizumab (Tysabri), fingolimod (Gilenya), and rituximab (Mabthera)] after January 1, 2002. We subsequently excluded patients younger than 18 years and older than 65 years at diagnosis (as they were not at risk for the study outcome, described below), patients with primary-progressive MS (due to the lack of standard treatment), and patients starting treatment after December 31, 2012 (to allow for a minimum of 1 year of follow-up and to avoid bias due to non-random censoring). Lastly, to facilitate interpretation of the analysis results (described below), patients were stratified into four pre-defined groups specified in terms of time from patient-reported onset of MS symptoms to initiation of first DMD treatment: (i) < 6 months, (ii) 6–12 months, (iii) 12–18 months, and (iv) ≥ 18 months.

We extracted patient-level inpatient comorbidity data from the Swedish National Patient Register, data on age, sex, migration, marital status, and education level from the Swedish Total Population Register and the Longitudinal Integration Database for Health Insurance and Labor Market Studies (LISA), and dates of death from the Swedish Cause of Death Register.

### Outcomes

The study outcome was defined as full-time (i.e., 100% of a full-time employment) disability pension. In Sweden, disability pension (i.e., sickness compensation, activity compensation, and early retirement pension) is granted to individuals who are unlikely to return to full-time employment due to disease or injury. We analyzed the outcome using claims data from the Swedish Social Insurance Agency’s database, Micro Data for Analysis of the Social Insurance (MiDAS) (data available up until December 31, 2013).

### Statistical analysis

We investigated the association between early treatment of MS and full-time disability pension using survival analysis. Specifically, we first estimated Kaplan–Meier failure functions with time measured from treatment initiation to full-time disability pension (i.e., the failure event). In the analyses, patients were right-censored for emigration, age 65 years, death, and date of last available record from the registries (December 31, 2013).

**Fig. 1:**
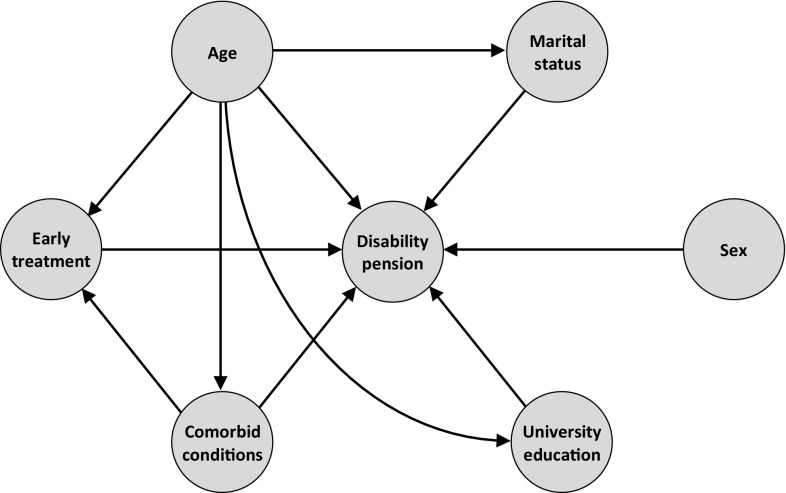
Directed acyclic graph of candidate variables. Early treatment refers to timing of treatment initiation in relation to onset of MS symptoms. Age, university education, and marital status were measured at treatment initiation; comorbid conditions (primary and secondary diagnosis, as specified in the International Statistical Classification of Diseases and Related Health Problems) were measured 5 years preceding treatment initiation

Next, we used regression analysis to explore predictors and derive adjusted estimates of the association between early treatment of MS and full-time disability pension. Candidates for explanatory variables included age (at treatment initiation), sex, marital status (at treatment initiation), university education (at treatment initiation), and prevalent comorbidities (primary and secondary diagnosis, as specified in the International Statistical Classification of Diseases and Related Health Problems (ICD-10), 5 years prior to the start date of the DMD treatment). The causal structure of the dependent and independent variables was examined using a directed acyclic graph (DAG) (Fig. 1). A DAG is a didactic framework for modeling relationships and causality and can be utilized to help inform, e.g., model specification in regression analysis to reduce the degree of bias for the effect estimate [14–16]. In brief, in a DAG, nodes represent variables (e.g., age and sex) and directed (single-headed) arrows direct causal effects; a chain is given by *A* → *D* → *Y* (i.e., *D* mediates the effect of *A* on *Y*), a fork is given by *A* ← *D* → *Y* (i.e., *D* is a common cause of both *A* and *Y*), and a collider (also known as inverted fork) is given by *A* → *D* ← *Y*. An introduction to DAGs are provided by Fleischer et al. [17].

We hypothesized that age at treatment initiation was a common cause of early treatment (negative relationship, as younger patients on average would be more likely to have initiated treatment early in relation to onset of disease symptoms) and disability pension (positive relationship, as younger patients would have had symptoms for a shorter duration of time, which in turn may be associated with, e.g., a lower prevalence of MS-related comorbidities, as well as a relatively high propensity to seek healthcare in general), and comorbidities (within 5 years of treatment initiation) to be a common cause of early treatment (positive relationship, as those with many comorbid conditions would be expected to seek care and initiate treatment earlier) and disability pension (positive relationship, as comorbid conditions would be expected to be associated with a higher general risk of disability pension). As a consequence, to obtain meaningful estimates of the association between early treatment and disability pension (for which we designated a causal path), both of these confounding variables should be included in the multivariable regression analysis. We did not identify any colliders that would impact (i.e., induce conditional dependence) with respect to the association between the exposure variable (i.e., early treatment) and outcome variable (i.e., disability pension) and therefore decided to include all candidate variables in the final regression model (specified in detail below).

In total, we fitted two Cox proportional hazards models. The first model (Model A) was specified to include time (in years) from onset of MS to treatment as a continuous variable to investigate if a significant association was present. The second model (Model B) was specified to include time from onset of MS as a categorical variable (four strata, as defined above), as well as age (at treatment initiation), sex, marital status (at treatment initiation), university education (at treatment initiation), and prevalent comorbidities (as defined above) in accordance with our DAG analysis, with the aim to explore predictors and control for confounding effects. We tested the proportional hazards assumption on the basis of Schoenfeld residuals.

We compared demographic and disease characteristics across the four categories of time from onset of MS to treatment using Welch’s analysis of variance and Pearson’s Chi-square test. All analyses were conducted in Stata 14.

## Results

A total of 2477 patients with MS initiated treatment with a DMD between January 1, 2002, and December 31, 2012 (maximum follow-up was 12 years; median 4 years). Table 1 presents summary characteristics of included patients, stratified by the four pre-defined strata of time from onset to treatment initiation. In the pooled sample, mean (SD) age at onset and diagnosis of MS was 33 (10) and 36 (10) years, respectively, 74% (1845 of 2477) were female, 41% (1016 of 2477) were married, and 43% (1055 of 2477) had a university education at treatment initiation.

**Table 1:** Summary characteristics of included patients (n = 2477)

	Time from onset of MS to treatment initiation				*p* value
	< 6 months	6–12 months	12–18 months	≥ 18 months	
*n* (proportion of total sample)	704 (28%)	407 (16%)	190 (8%)	1176 (47%)	
Age, mean (SD) years^a^	35 (9.8)	34.8 (9.8)	35.9 (9.5)	38.1 (9.6)	< 0.001
Sex, female	892 (76%)	134 (71%)	316 (78%)	503 (71%)	0.041
Married^a^	233 (33%)	150 (37%)	76 (40%)	557 (47%)	< 0.001
University education^a^	290 (41%)	156 (38%)	87 (46%)	522 (44%)	0.115
Follow-up, mean (SD) months	60.6 (36.5)	59.7 (35.3)	59.3 (34.9)	55.4 (33.8)	0.018
Time from onset to treatment initiation, mean (SD) months	3.1 (1.6)	8.7 (1.8)	14.8 (1.7)	80.3 (69.5)	< 0.001
Prevalent comorbidities^b^					
Certain infectious and parasitic diseases	28 (4%)	13 (3%)	7 (4%)	42 (4%)	0.924
Neoplasms	15 (2%)	12 (3%)	3 (2%)	33 (3%)	0.616
Diseases of the blood and blood-forming organs and certain disorders involving the immune mechanism	8 (1%)	4 (1%)	6 (3%)	9 (1%)	0.033
Endocrine, nutritional and metabolic diseases	38 (5%)	26 (6%)	14 (7%)	58 (5%)	0.450
Mental and behavioural disorders	26 (4%)	21 (5%)	12 (6%)	49 (4%)	0.358
Diseases of the nervous system^c^	225 (32%)	130 (32%)	39 (21%)	287 (24%)	< 0.001
Diseases of the eye and adnexa	81 (12%)	44 (11%)	16 (8%)	90 (8%)	0.028
Diseases of the ear and mastoid process	12 (2%)	5 (1%)	4 (2%)	33 (3%)	0.200
Diseases of the circulatory system	40 (6%)	18 (4%)	10 (5%)	60 (5%)	0.836
Diseases of the respiratory system	20 (3%)	22 (5%)	8 (4%)	38 (3%)	0.126
Diseases of the digestive system	44 (6%)	29 (7%)	11 (6%)	61 (5%)	0.504
Diseases of the skin and subcutaneous tissue	2 (0%)	4 (1%)	2 (1%)	11 (1%)	0.384
Diseases of the musculoskeletal system and connective tissue	33 (5%)	22 (5%)	11 (6%)	77 (7%)	0.404
Diseases of the genitourinary system	32 (5%)	21 (5%)	9 (5%)	62 (5%)	0.910
Symptoms, signs and abnormal clinical and laboratory findings, not elsewhere classified	157 (22%)	100 (25%)	44 (23%)	232 (20%)	0.169
Injury, poisoning and certain other consequences of external causes	28 (4%)	28 (7%)	10 (5%)	87 (7%)	0.023

Data presented as *n* (%) if not specified otherwise

^a^At treatment initiation

^b^Includes primary and secondary inpatient care diagnosis within 5 years of treatment initiation

^c^ Excluding diagnosis of multiple sclerosis

**Fig. 2:**
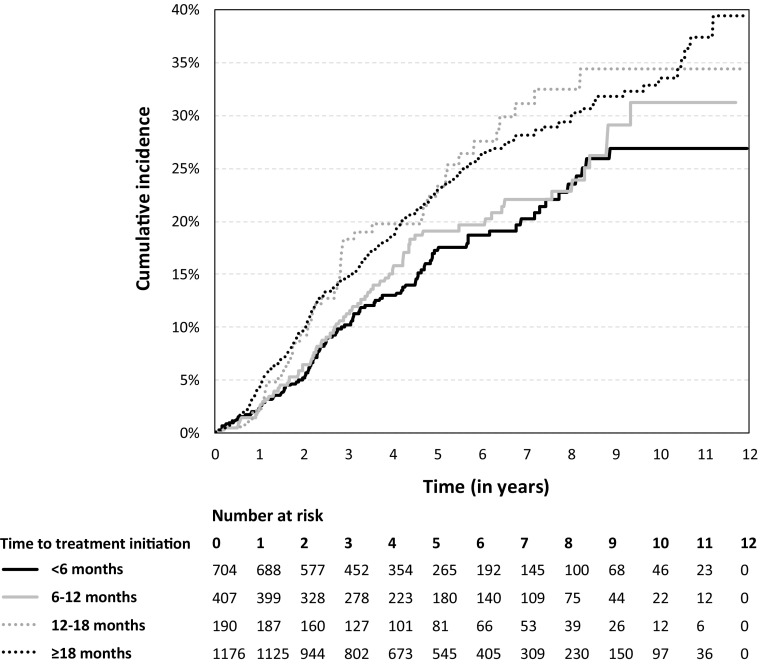
Cumulative incidence proportion of full-time disability pension

Figure 2 shows the unadjusted cumulative incidence proportion of full-time disability pension (100% of a full-time employment) after treatment initiation. Evident from the Kaplan–Meier failure functions, patients who started treatment within 6 months after onset (solid black line) had a markedly lower risk of disability pension across follow-up compared with patients initiating therapy after 12 months (dotted lines). Mean age at full-time disability pension was 40 years for patients who started treatment within 12 months, and 41 and 43 years for those who started within 12–18 months and after 18 months after disease onset, respectively. Complete life tables are available as supplemental material (Online Resource 1).

Results from the univariate Cox proportional hazards model (Model A) showed that time (in years) from onset of MS to treatment initiation was significantly associated with full-time disability (HR: 1.03, SD: 0.01, *p* < 0.001). Results from the multivariable Cox proportional hazards model (Model B) are presented in Table 2. Patients who started treatment within 6 months after onset were estimated to have, on average, a 36% lower risk of permanently leaving the labor force to receive full-time disability pension compared with a patient starting treatment after 18 months when adjusting for age, sex, marital status, university education, and the prevalence of comorbidities. A similar trend, although not statistically significant, was noted for patients who started treatment 6–12 months after onset. As expected, we also found age, sex, and university education to be significant determinants of full-time disability pension.

**Table 2:** Determinants of full-time disability pension (Cox proportional hazards model, Model B)

	Hazard ratio	Standard error	*p* value	95% CI
Time from onset to treatment initiation				
≥ 18 months (reference)	1			
12–18 months	1.11	0.18	0.521	0.81–1.52
6–12 months	0.78	0.11	0.074	0.59–1.02
< 6 months	0.74	0.09	0.010	0.58–0.93
Age, in years	1.03	0.01	< 0.001	1.02–1.05
Sex, female	1.34	0.15	0.009	1.08–1.67
Married	0.91	0.09	0.342	0.74–1.11
University education	0.53	0.05	< 0.001	0.44–0.64

Confidence interval (CI). Total sample: *n* = 2477. Age, marital status, and university education were measured at treatment initiation. Robust estimates were obtained using the Lin and Wei estimator of variance. The model was also adjusted for the prevalence of comorbidity diagnosis groups (as listed in Table 1) 5 years preceding treatment initiation. The proportional hazards assumption was tested on the basis of Schoenfeld residuals (global test *p* value > 0.05)

## Discussion

The objective of this study was to estimate the impact of early treatment on the risk of disability pension in patients with MS in Sweden. Our analyses show that early treatment with DMDs, as measured from onset of disease, was associated with a lower risk of full-time disability pension. Specifically, we found that patients who started treatment with a DMD within 6 months after disease onset on average had a 36% lower risk of full-time disability pension during follow-up treatment compared with patient starting treatment after 18 months. These findings highlight the potential benefits of early treatment of MS and should be helpful to inform ongoing discussion on the optimum medical management of the disease.

Comparing our results with previous research, several clinical trials have demonstrated a positive effect of early treatment of MS. For example, Goodin et al. [1, 2] found that interferon beta treatment at onset was associated with better physical and mental outcomes, as well as reduced mortality, compared with those receiving treatment after 3 years of placebo. Rovaris et al. [3] found similar evidence for patients treated with glatiramer acetate, Cocco et al. [4] for patients treated with DMDs, and the PRISMS Study Group and the University of British Columbia MS/MRI Analysis Group [5], Jacobs et al. [6], and Kappos et al. [7] for patients treated with interferon beta. More recently, in an observational study of Swedish patients with MS, Kavaliunas et al. [8] reported that patients had a 7% increased risk of reaching irreversible EDSS 4 for every year of delay in treatment start after MS onset. In this context, our study provides additional evidence that early treatment of MS may have a significant positive effect as studied outside the randomized controlled trial setting. Yet, additional research is needed to better understand the timing and impact of DMDs in the real-world clinical care of MS, in particular in the long term.

It should be noted that the association between early treatment and disability pension identified in our study may be subject to a phenomenon referred to by epidemiologists as confounding by indication. This bias arises when exposure (e.g., time from onset to treatment with DMDs) is associated (as a cause) with one or more patient characteristics (e.g., age) that in turn are associated (as a cause) with the outcome of interest (e.g., disability pension). Specifically, in our study, patients who initiated treatment early may have had more pronounced manifestations of disease activity, and thus been subject to a more aggressive disease compared with patients starting treatment later after onset. These differences would imply that patients who received treatment early would be expected to have less favourable health outcomes in the long term. Indeed, we found evidence for this in our data when comparing comorbidities between the four treatment groups, where, e.g., those who started treatment early (< 6 months) had significantly higher prevalence of diseases of the nervous system compared with patients who started treatment after 18 months (Table 1). A similar pattern would be expected to also be applicable to patients starting treatment 18 months after onset as compared to those who start later (after, e.g., 36 months). Therefore, with respect to this potential bias, our estimate of a 36% risk reduction of full-time disability pension should be interpreted as conservative. Moreover, in our analysis, we did not account for differences in treatment algorithms of MS over time in Sweden or compliance to treatment, both which may have influenced our estimates. This would be an interesting topic for future research to help obtain further insight of the association between early treatment and socio-economic outcomes, such as disability pension, for this disease population.

In agreement with previous research [12, 18–20], we found that patients with a university education had a significantly lower probability of full-time disability pension. A possible explanation for this finding is that patients without a university degree to a larger extent may be employed to perform blue-collar work characterized by, e.g., heavy lifts and strict work hours, which may be more difficult to maintain for a patient with MS. Another explanation could be that patients with a university education are more likely to seek healthcare and thus maintain healthy also in more advanced ages, a pattern identified in previous research of, e.g., myocardial infarction [21]. We also found age to be associated with an increased risk of disability pension, which would be expected to be a function of the natural evolution of MS, with increased disability and morbidity in higher ages, as well as the elevated morbidity profile of the elderly general population. Moreover, females were found to have a significantly higher risk of disability pension compared to men, a pattern also prevalent in the general Swedish population [22]. Possible reasons for this finding include gender segregation in the labour market in Sweden (where women to a larger extent than men have monotonous occupations that involve a high degree of repetitive strain with lower salary and limited involvement in decision-making) and fact that women on average carry a much larger burden of the work at home, which has been shown to be associated with impaired health [23].

A limitation of the present study concerns our inability to control for EDSS at treatment initiation due to lack of data. However, in a study of early treatment with DMDs on EDSS by Kavaliunas et al. [8], baseline EDSS was found to be similar for patients starting treatment within 1 year (mean: 1.6) and one to 3 years (mean: 1.6) after disease onset. This indicates that the impact of omitting this variable in our analysis may have been minor. Moreover, we did adjust the multivariable regression model for age, which we previously have shown to be a relatively robust proxy for disease progression in MS when analyzing medical absenteeism (i.e., sick leave and disability pension) [12]. A second limitation concerns our comorbidity data, which only comprised inpatient diagnoses. Consequently, the absolute levels of the reported conditions should be interpreted with caution. A third limitation concerns the retrospective nature of our data, which implies that we were unable to infer causality for the identified associations. Strengths of the present work include the relatively large sample of patients, the robust longitudinal datasets comprising both long-term clinical data from the Swedish Multiple Sclerosis Registry and demographic variables from several administrative databases in Sweden (covering the total Swedish population) which also allowed censoring for, e.g., death and emigration in our survival analysis.

In conclusion, we show that early treatment with DMDs of MS is associated with a significantly reduced risk of disability pension. These findings should help inform therapeutic decisions in MS. Yet, more research is needed to fully understand the timing of treatment with DMDs on long-term health and socio-economic outcomes in MS.

## Electronic supplementary material

Below is the link to the electronic supplementary material.

Supplementary material 1 (DOCX 16 kb)
