# Supplementary material for: The long-term impact of early treatment of multiple sclerosis on the risk of disability pension
Source: J Neurol. 2018 Feb 1;265(3):701–7. doi: 10.1007/s00415-018-8764-4 (PMC5834562; doi:10.1007/s00415-018-8764-4)
Supplement: Supplementary file 1 — Supplementary material 1 (DOCX 16 kb) [file 415_2018_8764_MOESM1_ESM.docx]

**Online Resource 1**

**Table e-1: Life table for patients starting treatment <6 months after onset**

| **Time point (years)** | **Beginning** | **Fail** | **Failure function** | **SE** | **95% CI** |
| --- | --- | --- | --- | --- | --- |
| 0 | 704 |  |  |  |  |
| 1 | 688 | 16 | 0.023 | 0.006 | 0.014-0.037 |
| 2 | 577 | 19 | 0.052 | 0.009 | 0.037-0.071 |
| 3 | 452 | 29 | 0.102 | 0.012 | 0.081-0.129 |
| 4 | 354 | 13 | 0.130 | 0.014 | 0.105-0.160 |
| 5 | 265 | 15 | 0.172 | 0.017 | 0.142-0.209 |
| 6 | 192 | 4 | 0.187 | 0.018 | 0.154-0.226 |
| 7 | 145 | 3 | 0.202 | 0.020 | 0.166-0.245 |
| 8 | 100 | 5 | 0.235 | 0.024 | 0.192-0.286 |
| 9 | 68 | 4 | 0.269 | 0.029 | 0.218-0.330 |
| 10 | 46 | 0 | 0.269 | 0.029 | 0.218-0.330 |
| 11 | 23 | 0 | 0.269 | 0.029 | 0.218-0.330 |

**Table e-2: Life table for patients starting treatment 6-12 months after onset**

| **Time point (years)** | **Beginning** | **Fail** | **Failure function** | **SE** | **95% CI** |
| --- | --- | --- | --- | --- | --- |
| 0 | 407 |  |  |  |  |
| 1 | 399 | 9 | 0.022 | 0.007 | 0.012-0.042 |
| 2 | 328 | 16 | 0.064 | 0.013 | 0.044-0.094 |
| 3 | 278 | 16 | 0.113 | 0.017 | 0.084-0.150 |
| 4 | 223 | 12 | 0.154 | 0.020 | 0.120-0.198 |
| 5 | 180 | 9 | 0.191 | 0.022 | 0.152-0.240 |
| 6 | 140 | 1 | 0.196 | 0.023 | 0.156-0.246 |
| 7 | 109 | 4 | 0.220 | 0.025 | 0.176-0.275 |
| 8 | 75 | 1 | 0.229 | 0.026 | 0.182-0.285 |
| 9 | 44 | 5 | 0.291 | 0.036 | 0.227-0.369 |
| 10 | 22 | 1 | 0.312 | 0.041 | 0.240-0.400 |
| 11 | 12 | 0 | 0.312 | 0.041 | 0.240-0.400 |

**Table e-3: Life table for patients starting treatment 12-18 months after onset**

| **Time point (years)** | **Beginning** | **Fail** | **Failure function** | **SE** | **95% CI** |
| --- | --- | --- | --- | --- | --- |
| 0 | 190 |  |  |  |  |
| 1 | 187 | 4 | 0.021 | 0.010 | 0.008-0.055 |
| 2 | 160 | 13 | 0.092 | 0.021 | 0.058-0.144 |
| 3 | 127 | 15 | 0.183 | 0.030 | 0.133-0.250 |
| 4 | 101 | 2 | 0.197 | 0.031 | 0.145-0.266 |
| 5 | 81 | 4 | 0.234 | 0.034 | 0.174-0.309 |
| 6 | 66 | 4 | 0.275 | 0.038 | 0.208-0.358 |
| 7 | 53 | 3 | 0.311 | 0.042 | 0.238-0.401 |
| 8 | 39 | 1 | 0.325 | 0.043 | 0.249-0.417 |
| 9 | 26 | 1 | 0.344 | 0.046 | 0.263-0.442 |
| 10 | 12 | 0 | 0.344 | 0.046 | 0.263-0.442 |
| 11 | 6 | 0 | 0.344 | 0.046 | 0.263-0.442 |

**Table e-4: Life table for patients starting treatment ≥18 months after onset**

| **Time point (years)** | **Beginning** | **Fail** | **Failure function** | **SE** | **95% CI** |
| --- | --- | --- | --- | --- | --- |
| 0 | 1,176 |  |  |  |  |
| 1 | 1,125 | 50 | 0.043 | 0.006 | 0.032-0.056 |
| 2 | 944 | 61 | 0.097 | 0.009 | 0.081-0.116 |
| 3 | 802 | 51 | 0.148 | 0.011 | 0.128-0.170 |
| 4 | 673 | 35 | 0.187 | 0.012 | 0.165-0.213 |
| 5 | 545 | 33 | 0.230 | 0.014 | 0.204-0.258 |
| 6 | 405 | 21 | 0.263 | 0.015 | 0.236-0.294 |
| 7 | 309 | 9 | 0.281 | 0.016 | 0.252-0.313 |
| 8 | 230 | 6 | 0.297 | 0.017 | 0.266-0.331 |
| 9 | 150 | 6 | 0.318 | 0.018 | 0.284-0.355 |
| 10 | 97 | 3 | 0.336 | 0.020 | 0.297-0.377 |
| 11 | 36 | 4 | 0.374 | 0.027 | 0.324-0.429 |
